# Systematic Review on Large Language Models in Orthopaedic Surgery

**DOI:** 10.3390/jcm14165876

**Published:** 2025-08-20

**Authors:** Kevin Mo, Rowen Lin, Evan Dunn, Gio Girgis, William Fang, John Walsh, Nicole Banyai-Flores, Troy Watson, Daniel Lee

**Affiliations:** 1 Orthopaedic Surgery, Valley Hospital Medical Center, 620 Shadow Ln, Las Vegas, NV 89106, USA; kevinchowahmo@gmail.com (K.M.); thewillfang@gmail.com (W.F.); walshjohn2014@gmail.com (J.W.); 2 Touro University Nevada College of Osteopathic Medicine, 874 American Pacific Dr, Henderson, NV 89104, USA; rlin6@student.touro.edu (R.L.); ggirgis@student.touro.edu (G.G.); 3 Desert Orthopaedic Center, 2800 E. Desert Inn Rd, Las Vegas, NV 89121, USA; nicolesabrina0320@gmail.com (N.B.-F.); teamwatson@doclv.com (T.W.); dlee@doclv.com (D.L.)

**Keywords:** large language models, orthopaedic surgery, ChatGPT, systematic review

## Abstract

**Background/Objectives**: Since ChatGPT was released in 2022, many Large Language Models (LLM) have been developed, showing potential to expand the field of orthopaedic surgery. This is the first systematic review looking at the current state of research of LLMs in orthopaedic surgery. The aim of this study is to identify which LLMs are researched, assess their functionalities, and evaluate their quality of results. **Methods**: The systematic review was conducted using PubMed, Embase, and Cochrane Library databases in accordance with Preferred Reporting Items for Systematic Reviews and Meta-Analyses (PRISMA) guidelines. **Results**: A total of 60 studies were included in the final review, all of which included ChatGPT versions 3.0 or 4.0. There were five studies that included Bard and one article each for Perplexity AI and Bing. Most studies assessed orthopaedic assessment questions (23 studies) and their ability to correctly answer free ended questions (31 studies). Outcome measures used to assess the accuracy of LLMs in most of the included studies were the percentage of correct answers on multiple-choice questions or expert-graded consensus to open-ended responses. The accuracy of ChatGPT 4.0 in orthopaedic assessment questions ranged from 47.2 to 73.6% without images, and 35.7–65.85% with images. The accuracy of ChatGPT 3.5 was 29.4–55.8% without images and 22.4–46.34% with images. The accuracy of Bard ranged from 49.8 to 58%. Orthopaedic residents consistently scored better than LLMs in the range of 74.2–75.3%. **Conclusions**: ChatGPT 4 showed significant improvement over ChatGPT 3.5 in answering orthopaedic assessment questions. When comparing performances of orthopaedic residents to LLMs, orthopaedic residents scored higher overall. There remains significant opportunity for development of LLM performance on orthopaedic assessments as well as image-based analysis and clinical documentation.

## 1. Introduction

Large language models are a type of artificial intelligence model that can process, organize, and generate human language in an understandable and meaningful way. ChatGPT, an LLM released to the public in November 2022 by OpenAI, represented a significant leap forward in the field of artificial intelligence due to its human-like conversational abilities, accessibility, and vast amount of computational data and parameters. For example, from the moment it was released to the public, ChatGPT was immediately able to generate creative content such as stories, poems, and songs; generate computer programming code; emulate conversational styles and humour; and learn from human feedback. Since then, multiple LLMs have been released by competing technology companies including Google’s Bard and Perplexity AI backed by Amazon’s Jeff Bezos and NVIDIA [1,2]. LLMs have demonstrated across multiple disciplines that they are able to perform at par or above the abilities of humans, perform general functions, communicate, and conduct data analysis [3]. Since their advent, studies of LLMs in various subspecialties of medicine, including orthopaedic surgery, have gained significant traction.

Odabashian et al. studied ChatGPT 3.5’s ability to assess oncology questions [4]. There is also literature indicating that LLMs performed relatively well in dermatology and neurology board examinations, meeting the threshold to pass [5,6]. LLMs have also been assessed for their ability in radiology and ophthalmology examinations; however, they did not meet the threshold to pass such exams [7,8]. To summarize, LLMs have shown the capability of performing well and possibly passing medical board examinations across multiple subspecialties.

LLMs in orthopaedic surgery have many theoretical opportunities to improve patient care. In the triage of orthopaedic injuries in the Emergency Room (ER), LLMs can be used for clinical decision support to assist in the analysis of patient data such as imaging, lab results, and documentation to suggest differential diagnoses and treatment algorithms [9,10,11]. LLMs have also been explored for generating patient educational content tailored to specific learners’ levels, have shown proficiency on orthopaedic in-training exams, and have demonstrated some ability to interpret clinical imaging such as X-ray and Computed Tomography (CT) [11,12]. Despite the growing interest within the field of orthopaedics, synthesized research on LLMs remains limited.

To our knowledge, no systematic review has been conducted in the literature assessing the recent research on LLMs’ performance in orthopaedic surgery since their advent in 2022. The aim of our study is to assess current studies of LLMs in orthopaedic surgery in order to identify current areas of research, trends in publication, as well as the evolving performance of LLMs.

## 2. Materials and Methods

This systematic review was conducted in accordance with the Preferred Reporting Items for Systematic Reviews and Meta-Analyses (PRISMA) [13]. The study was exempt from Institutional Review Board approval. A comprehensive literature search was conducted through multiple databases on 1 May 2024 to identify articles relevant to the assessment of Large Language Models. Our search strategy comprised a combination of key terms including: “Large language model”, “ChatGPT”, “ChatGPT 3.0”, “ChatGPT 3.5”, “ChatGPT 4.0”, “Bard”, “orthopaedic surgery”, “orthopaedic”, “orthopedic”, “orthopedic surgery”.

Articles were screened based on the following inclusion criteria: 1. full-length text studies, 2. published or translated into English, 3. analysis of the capabilities of Large Language Models in orthopaedics. Studies were excluded from this systematic review if they were: 1. non-English articles, 2. articles without available full texts, 3. non-relevant studies.

Two reviewers performed the electronic database search through PubMed, Embase, and Cochrane Library databases. Articles were then independently reviewed by each reviewer to assess if they would be included in the final review. Disagreements were resolved by consensus or by consultation with a third reviewer. Manual searches through references and bibliographies of included studies were conducted to find relevant articles not shown in the electronic database searches.

The following data points were recorded from the articles included in our review: year of publication, journal of publication, type of LLMs studied, capability of LLM assessed. When applicable, the following additional variables were also assessed: accuracy of LLM versus orthopaedic residents in exam questions, performance of LLM in image versus text questions, comparison performance in different LLM versions, scales used to assess LLM responses, accuracy of LLM responses to common patient questions.

Formal risk of bias and certainty assessments were not conducted, as the included studies varied substantially in their designs and methodologies. This heterogeneity limits the interpretability of standard assessment tools, and their use could provide misleading conclusions. The primary goal of this review was to synthesize current literature, rather than assess causal effects. This systematic review was not prospectively registered in a publicly accessible database, and no formal protocol was prepared.

## 3. Results

### 3.1. Study Selection

**Figure 1:**
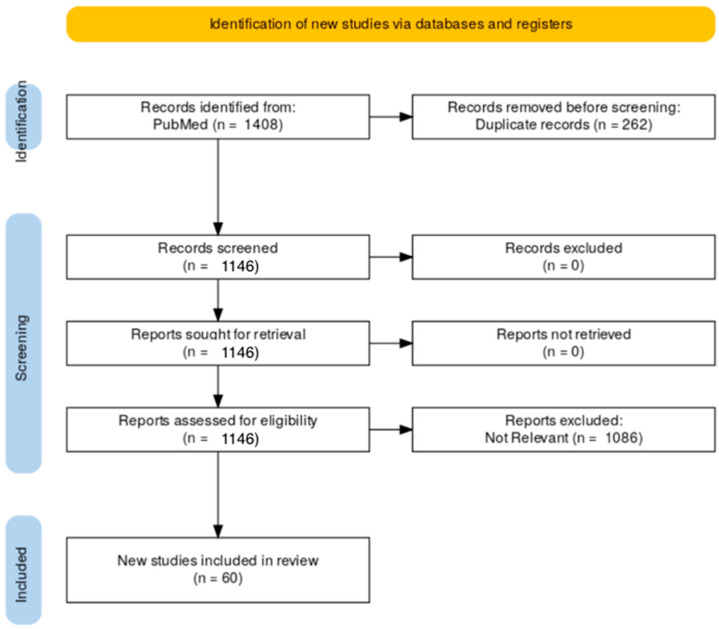
Preferred reporting items for systematic reviews and meta-analyses flow diagram of literature results and screening.

Our comprehensive systematic search yielded 1408 total articles. Of these studies, 262 articles were excluded for being duplicates. Abstracts and titles of the remaining 1146 studies were then assessed for eligibility. After applying our inclusion and exclusion criteria, 1086 articles were removed, leaving a final total of 60 articles. Figure 1 shows the PRISMA search strategy.

### 3.2. Study Characteristics

**Figure 2:**
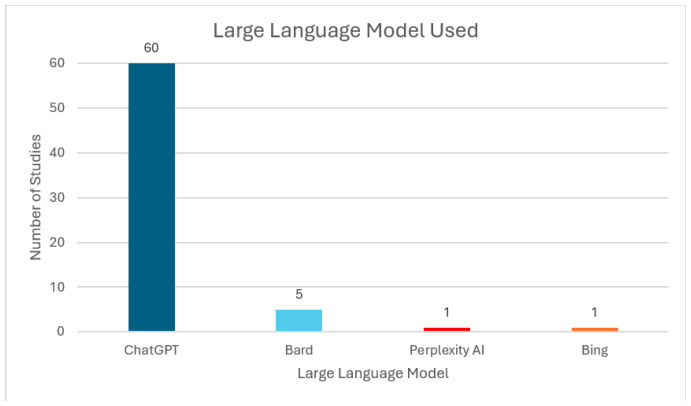
Chart showing the type of Large Language Models studied in medical literature for orthopaedics. For each type, the number of studies using that Large Language Model is represented by the vertical axis.

**Figure 3:**
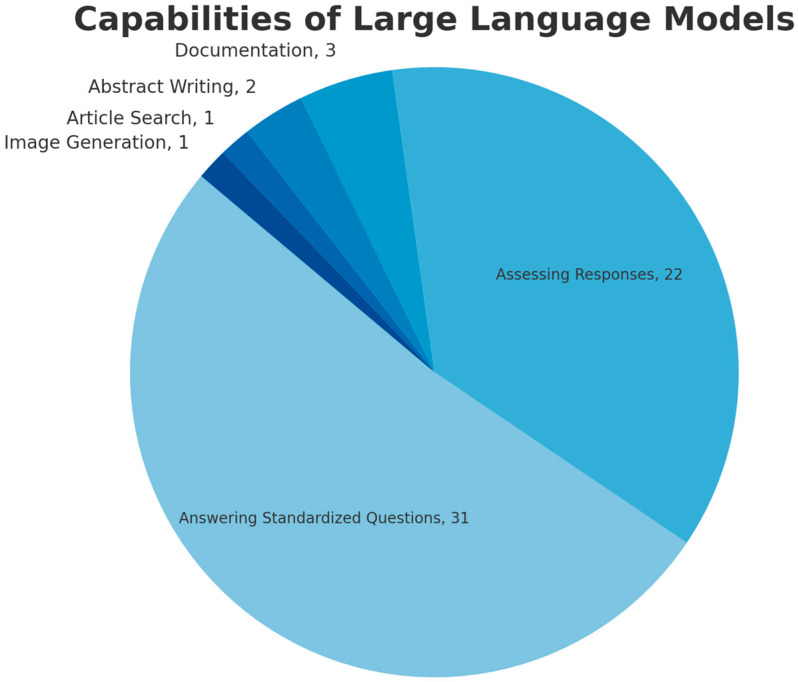
Pie chart demonstrating the capabilities of Large Language Models commonly studied. For each capability, the number of studies reporting it is included as a pie chart label.

Figure 2 shows the number of studies that analyzed each type of LLM. Out of the 60 articles identified, all 60 included ChatGPT, 5 articles investigated Bard (8.3%), 1 article used Perplexity AI, and 1 article used Bing, which runs on GPT (1.6%). Figure 3 shows the capabilities of the LLMs that each study investigated. Assessing the LLMs’ accuracy in answering standardized general orthopaedic questions was the most commonly performed study with 31 articles (51.7%), followed by 22 studies assessing the LLMs’ free-text response to common patient questions or clinical scenarios (36.7%). Most of the standardized multiple-choice questions utilized in these studies were derived from orthopaedic in-training examinations or board-style assessments, including questions from validated sources such as OrthoBullets and the American Board of Orthopaedic Surgery (ABOS). There were relatively fewer articles assessing the LLMs’ efficacy in abstract writing (2), documentation ability (3), article searching (1), and image generation (1).

### 3.3. LLM Capability in Answering Standardized Exam Questions

Supplemental Table S1 demonstrates six studies directly comparing ChatGPT 3.5 to 4.0 and two studies comparing ChatGPT to Bard [14,15,16,17,18,19,20]. The accuracy of LLMs’ answers to standardized orthopaedic assessment questions was recorded in 23 studies. When assessed specifically without the utilization of images in standardized questions, ChatGPT 3.5 showed an accuracy of 18–60.8%. With the incorporation of images, accuracy was significantly reduced to 22.4–46.34%, with an overall accuracy rate of 29.4–55.8%. ChatGPT 4.0 performed better than ChatGPT 3.0 and 3.5, achieving an accuracy rate of 61–73.6% when no images were included. Several studies reported this difference as statistically significant (*p* = 0.002 [14]; *p* < 0.001 [17]; *p* = 0.268 [21]). When images were included, ChatGPT 4.0 showed an accuracy rate of 35.7–65.85%, with an overall accuracy of 47.2–73.6%. Again, multiple studies exhibited statistically significant differences in the performance of these LLM versions (*p* = 0.002 [15]; *p* < 0.001 [14]; *p* < 0.00001 [22]).

Two studies compared ChatGPT to Bard as well as ChatGPT to orthopaedic residents’ performance. Bard recorded an overall accuracy of 49.8–58% and the orthopaedic residents scored 74.2–75.3% overall. Orthopaedic residents performed better than both versions of ChatGPT in all studies included in this review (*p* < 0.001; *p* = 0.044 ChatGPT 3.5; *p* = 0.019 ChatGPT 4.0) [15,21].

Table 1 includes seven articles that compared LLM efficacy with images versus without images. Within these articles, ChatGPT performed either the same or better (18–73%), than with the use of images (22.4–65.85%) (*p* = 0.416; *p* = 0.155; *p* < 0.001; *p* = 0.033 ChatGPT 3.5; *p* < 0.001 ChatGPT 4.0) [15,17,23,24].

### 3.4. ChatGPT 3.5 vs. ChatGPT 4.0 vs. Bard

Table 2 includes the articles comparing ChatGPT and Bard. ChatGPT was the most common LLM investigated. Bard was the only other LLM analyzed by multiple studies, with four articles comparing the performance of Bard to ChatGPT [18,19,25,26]. Two articles showcased Bard’s accuracy in answering standardized questions relative to ChatGPT. Lum et al. showed that Bard actually performed better overall at 58% compared to 47% [18]. However, Lubitz et al. showed conflicting results with Bard performing significantly worse at 49.8% overall and 58% without images versus ChatGPT’s performance of 69.1% overall and 77.8% without images (*p* < 0.0001) [19].

Two articles also analyzed both LLMs’ performances in answering patient questions and questions through OrthoBullets [25,26]. ChatGPT performed superiorly, aligning with the guidelines of the American Orthopaedic Foot and Ankle Society at 46.2% versus Bard at 36.5%. However, Agharia et al. showed conflicting results, where Bard performed better in picking the more popular answers in OrthoBullets at 45.4% compared to ChatGPT 3.5 at 40.2%. ChatGPT 4.0 outperformed both at an accuracy rate of 68% (*p* < 0.001) [26].

Supplemental Table S2 includes the articles that compare ChatGPT 3.5 and ChatGPT 4.0 [26,27,28,29]. ChatGPT 4.0 shows significant differences from ChatGPT 3.5 in ability to answer standardized orthopaedic questions as shown in Table 1. Both Mejia et al. and Zaidat et al. compared ChatGPT’s accuracy to North American Spine Society (NASS) guidelines [28,29]. ChatGPT 4.0 showed an accuracy to NASS guidelines of 59–81%, with 19–28% of its responses being incomplete. ChatGPT 3.5 in comparison had an accuracy of 52–62.5% to NASS guidelines, an incomplete response being recorded 37.5–38% of the time. Fahy et al. showed that ChatGPT 4.0 not only demonstrated a higher score on the DISCERN scale (62.09 vs. 55.4) but also a lower score on the Flesch-Kincaid scale (13.7 vs. 14.7), indicating its ability to simplify information by a grade level [27].

### 3.5. LLM Capability in Answering General Patient Questions

The main capability of LLMs that was investigated was their accuracy in answering general patient questions regarding orthopaedic treatment modalities. As shown in Table 3, the most common method of assessment was for physicians to grade the LLM’s response (17 articles, 44.7%). Various other scales were utilized including the Likert, DISCERN, and Flesch-Kincaid scales (four, five, and six articles, respectively). Another method used was comparison of LLM’s response to recommended orthopaedic treatment guidelines (6 articles, 15.7%).

Multiple scales were used to assess the response of LLMs to patient questions, with Likert, DISCERN, and Flesch-Kincaid being the most commonly utilized. In Supplemental Table S3, ChatGPT was shown to have a Likert score from 3.87/5–4.9/5, a DISCERN score of 41–62.09, and Flesch-Kincaid scores of 11.2–26.2. The Likert scores in these studies demonstrated that the evaluators deemed ChatGPT’s responses to be satisfactory. The DISCERN scores depicted its responses to be in a range from fair to just shy of excellent. The Flesch-Kincaid scores demonstrated the range of complexity of the ChatGPT responses to be from a high school to post-graduate level.

In terms of comparison, Fahy et al. showed ChatGPT 4.0 obtained a higher DISCERN score than ChatGPT 3.5 (62.09 vs. 55.4; *p* < 0.01) as well as a negligible difference in Flesch-Kincaid scores (17.9 vs. 18.08; *p* = 0.95) [27]. Supplemental Table S3 also shows two articles comparing ChatGPT 3.5 and 4.0 to NASS guidelines, demonstrating ChatGPT 4.0 to have a higher accuracy (58.6–81.25%) versus ChatGPT 3.5 (51.7–62.5%) [28,29]. Yang et al. looked at the quality of recommendations generated by ChatGPT and Bard based on their concordance with the American Academy of Orthopaedic Surgeons (AAOS) clinical practice guidelines. ChatGPT’s responses were concordant with guidelines in 80% of responses, compared to 60% for Bard. Of note, ChatGPT suggested treatments that were not recommended by AAOS in 30% of responses, while Bard suggested non-recommended treatments in 60%. Only one study compared Perplexity AI and Bing, respectively, which limited the opportunity for meaningful comparison. While our review found that these models generally performed at lower accuracy rates than ChatGPT, this observation should be interpreted with caution. Only a small number of studies evaluated these alternative models, and few conducted direct testing between Perplexity AI, Bing, and ChatGPT under identical conditions. Thus, these results reflect a description tending in the current literature, rather than a statistically robust comparative analysis of these lesser-examined LLMs.

## 4. Discussion

The aim of this study was to assess the current state of research on LLMs in orthopaedic surgery. The LLMs included in this systematic review were ChatGPT (3.0, 3.5, and 4.0), Bard, Bing, and Perplexity AI. The vast majority of studies utilized ChatGPT and assessed the LLMs’ ability to perform either on orthopaedic in-training examination or on common patient questions. LLMs overall performed at a level lower than orthopaedic surgery trainees on in-training exams [15,21]. Newer LLMs performed relatively better than their older counterparts [14,15,16,17,20,21,22,26,27,29].

Regarding orthopaedic in-training examinations, LLMs have shown the capability of answering questions across multiple studies in our review, with an accuracy range of 22.4% to 77.8%. However, results vary depending on the version of LLM and whether the LLM was given images or no images, as seen in Table 1 and Table S2. Interestingly, LLMs seemed to perform worse when images were included for analysis. This may be due to the current versions’ limited ability to evaluate visual data. These multimodal models have the capability to interpret images, but their development has historically been text-focused, resulting in diminished performance in tasks requiring visual reasoning [14,17,20,21,22]. In addition, the evaluation of orthopaedic imaging often requires specific pattern recognition skills that are seemingly not fully reproducible by current LLM. The findings synthesized in this review suggest that further improvements in training and integration of visual processing models by LLMs are required.

This review included a total of 22 studies that assessed LLMs’ ability to answer patients’ questions. Our review has shown promising results of LLMs, most prominently ChatGPT, performing near the same level as trained healthcare providers in answering general patient questions. The accuracy of the LLM was determined by physicians’ assessment or by the guidelines Likert, DISCERN, and Flesch-Kincaid. Liu et al. have found that LLMs perform at an adequate level of answering patients’ messages in their patient portals to a similar level as physicians [30]. A study comparing the responses of LLMs to ophthalmologists; answers to patient questions found comparable error rates, with little difference between the answers of LLMs and physicians [31]. Additional findings in the literature show LLMs performing comparably well to physicians regarding benign prostate hyperplasia and radiation oncology patient questions [32,33]. These findings highlight the possibility of a future where LLMs and AI alike may be utilized by physicians and healthcare providers to save time, improve efficiency, and meet patients’ needs. It is important to note that LLMs are still relatively new and rapidly evolving, and current studies may not be representative of the future. Nevertheless, these early findings of LLMs performing well in answering patient questions are promising, and we may expect better results and more studies as the field of Artificial Intelligence (AI) and LLMs continues to evolve.

Large Language Models have made large improvements over recent years, consistently releasing new versions such as ChatGPT 4.0. Relative to ChatGPT 3.5, Version 4.0 demonstrates a remarkably improved accuracy to standardized questions and improved quality of response to general patient questions. Performance has been enhanced not just in orthopaedics but other subspecialties as well, including dermatology and paediatric cardiology, with the ability to pass board examinations [34,35]. It is important to note that while ChatGPT 4.0 is the newest version, version 5.0 is due for release soon. Sam Altman, the CEO of OpenAI, in an interview with *The Lex Fridman Podcast*, stated that ChatGPT 4.0 is much worse than the new upcoming version 5.0, “I expect the delta between 5 and 4 will be the same as between 4 and 3. It’s our job to live in the future and remember that our tools are going to kind of suck looking back at them” [36].

This article has multiple limitations. Firstly, large language models are updating and developing constantly; thus, the performance of a large language model may be different from month to month even within the same version. Furthermore, there was limited investigation into the performance of other models such as Bard, Bing, or Perplexity AI, constraining the ability to perform a quantitative subgroup analysis depending on the LLM used. The ability of large language models to answer standardized questions as well as patient questions regarding medicine-related questions appears to be the most researched aspect, with limited research into their ability for image recognition, image generation, and documentation. The studies included in this review demonstrated a reduced ability to answer orthopaedic questions when visual reasoning or image recognition was included, compared to text-only inquiries. This finding illustrates the preference in current LLMs for language processing rather than in-depth visual evaluation. Future adaptations to address these issues within the field of orthopaedics could include the incorporation of specific vision models trained from large orthopaedic datasets, co-training models on paired imaging and expert-dictated reports, and eventually focusing on fine-tuning by using subspecialty-specific imaging libraries. These limitations indicate that future research should not only investigate the endless capabilities of these large language models but also compare ChatGPT to its competitors. Standardized, direct comparative evaluations are required before a definitive decision can be made on which LLM is most accurate within the realm of orthopaedic surgery.

## 5. Conclusions

The majority of studies in this systematic review were performed from early 2023 to mid-2024, utilized ChatGPT, and assessed LLM performance in orthopaedic board questions or common patient questions. ChatGPT has yet to surpass orthopaedic surgery residents in performance on in-training examination questions. Overall, newer LLMs performed better than their predecessors. Many functionalities of LLMs—such as image analysis, clinical documentation generation, and the capabilities of newer models like GPT 4o—were not studied and represent key opportunities for future research.

## Figures and Tables

**Table 1:** LLM performance with vs. without images.

Study Name	ChatGPT Version	With Images	Without Images	*p*-Value
Massey et al. 2023 [15]	3.5	22.4%	37.8%	0.033
	4.0	35.7%	61.0%	<0.001
Ghanem et al. 2023 [24]	4.0	57.6%	64.2%	0.416
Ghanem et al. 2024 [23]	4.0	55.56%	65.83%	0.155
Rizzo et al. 2024 [20]	4.0	65.85%	68.8%	-
	4.0	51.92%	65.14%	
	4.0	46.51%	68.22%	
	3.5	46.34%	52.80%	
	3.5	42.31%	52.29%	
	3.5	38.32%	51.94%	
Nakajima et al. 2024 [14]	3.5	28, 32, 30%	33, 27, 18%	-
	4.0	60, 55, 61%	64, 63, 73%	
Posner et al. 2024 [17]	4.0	47.59%	67.81%	<0.001 overall
Fielder et al. 2024 [21]	4.0	53.2%	66.7%	-

**Table 2:** ChatGPT vs. Bard.

Study Name	ChatGPT	Bard	*p*-Value
Lum 2023 [18]	Difficulty Level 1: 54% Difficulty Level 2: 51% Difficulty Level 3: 34% Overall: 47%	Overall: 58%	-
Lubitz et al. 2024 [19]	Overall: 69.1% Text describing media: 77.8%	Overall: 49.8% Text describing media: 58%	*p* < 0.0001 (overall &text describing media)
Parekh et al. 2024 [25]	AOFAS patient education: 46.2%	AOFAS patient education: 36.5%	-
Agharia et al. 2023 [26]	ChatGPT 3.5 (popular response in OrthoBullets): 40.2% ChatGPT 4.0: 68.0%	Popular response in OrthoBullets: 45.4%	*p* < 0.001

**Table 3:** Assessment criteria for LLM response.

Assessment Criteria	Number of Studies
Assessed by Physician	17
Likert	4
DISCERN	5
Flesch-Kincaid	6
Accuracy to Recommended Guidelines	6

## Data Availability

Data is contained within the article or Supplementary Material. Further inquiries can be directed to the corresponding author(s).

## Supplementary Materials

The following supporting information can be downloaded at: https://www.mdpi.com/article/10.3390/jcm14165876/s1. Table S1: Accuracy of LLM Answers to Orthopaedic Assessment Questions versus Orthopaedic Residents; Table S2: Comparison of ChatGPT 3.5 vs. ChatGPT 4.0; Table S3: LLM performance on Patient Questions.

## Abbreviations

The following abbreviations are used in this manuscript:

LLM	Large Language Models
PRISMA	Preferred Reporting Items for Systematic Reviews and Meta-Analyses
ER	Emergency Room
CT	Computed Tomography
ABOS	American Board of Orthopaedic Surgery
AI	Artificial Intelligence
NASS	North American Spine Society
AAOS	American Academy of Orthopaedic Surgeons
