# Supplementary material for: Systematic Review on Large Language Models in Orthopaedic Surgery
Source: J Clin Med. 2025 Aug 20;14(16):5876. doi: 10.3390/jcm14165876 (PMC12386971; doi:10.3390/jcm14165876)
Supplement: Supplementary file 1 [file jcm-14-05876-s001.zip › LLM Supplemental Table S1.pdf]

Study Name:	ChatGPT unspecified	ChatGPT 3.5	ChatGPT 4.0	Bard	Orthopaedic Residents	p-values
Massey et al. 2023	-	37.8 % (no images)<br>22.4% (with images)<br>29.4% (overall)	61.0% (no images)<br>35.7% (with images)<br>47.2% (overall)	-	74.2% (overall)	p<0.001<br>p<0.001 (3.5/4.0 vs Residents)<br>p=0.002 (4.0 vs 3.5)
Kung et al. 2023	-	54.3% (overall no images)	73.6% (overall no images)	-	-	-
Lum. 2023	54% (Tax 1)<br>51% (Tax 2)<br>34% (Tax 3)<br>47% (overall)	-	-	58% (overall)	-	p=0.034 (overall)
Ghanem et al. 2023	-	-	64.2% (no images)<br>57.6% (with images)<br>61.2% (overall)	-	-	p = 0.416 for OITE questions with media
Jain et al. 2024	-	55.8% (OITE 2020)<br>47.7% (OITE 2021)<br>54% (OITE 2022)	-	-	-	Significant associations found between test topic and correct answer (p = 0.011), and type of logic used and tested topic p = < 0.001
Saad et al. 2023	-	-	67.5% (overall)<br>50% (Part 1)<br>85% (Part 2)	-	-	-
Ghanem et al. 2024	-	-	55.56% (with media)<br>65.83% (without media)<br>61.98% (overall)	-	-	-
Rizzo et al. 2023	-	50.24% (2022 overall)<br>47.42% (2021 overall)<br>46.51% (2020 overall)<br>46.34% (2022 with media)<br>42.31% (2021 with media)<br>38.37% (2020 with media)<br>52.80% (2022 without media)<br>52.29% (2021 without media)<br>51.94% (2020 without media)<br>38.30% (2022 first order)<br>50.00% (2021 first order)<br>53.70% (2020 first order)<br>53.75% (2022 higher order)	67.63% (2022 overall)<br>58.69% (2021 overall)<br>59.53% (2020 overall)<br>65.85% (2022 with media)<br>51.92% (2021 with media)<br>46.51% (2020 with media)<br>68.8% (2022 without media)<br>65.14% (2021 without media)<br>68.22% (2020 without media)<br>63.83% (2022 first order)<br>57.45% (2021 first order)<br>65.74% (2020 first order)<br>68.75% (2022 higher order)<br>59.66% (2021 higher order)<br>53.27% (2020 higher order)	-	-	-

		45.38% (2021 higher order)<br>39.25% (2020 higher order)				
Isleem et al. 2023	60.8% (overall)	-	-	-	-	-
Nakajima et al. 2024	-	28, 32, 30% (all questions)<br>33, 27, 18% (without images)<br>30% (overall)	60, 55, 61% (all questions)<br>64, 63, 73% (without images)<br>59% (overall)	-	-	p < 0.001 (4.0 vs 3.5 all questions)<br>p=0.002 (text based)
Lum et al. 2024	54% (overall)	-	-	-	-	-
Posner et al. 2024	-	53.4% (overall 1st entry)<br>50.1% (overall 2nd entry)	59.36% (overall 1st entry)<br>56.60% (overall 2nd entry)<br>67.81% (no images)<br>47.59% (with images)	-	-	p<0.001 (4.0 vs 3.5 with no images)<br><br>p=0.947 (1st vs 2nd entry for images on 4.0)<br><br>p=0.226 (2nd entry 4.0)<br><br>p=0.305 (2nd entry 3.5)
Posner et al. 2024	-	53.4% (overall 1st entry)<br>50.1% (overall 2nd entry)	59.36% (overall 1st entry)<br>56.60% (overall 2nd entry)<br>67.81% (no images)<br>47.59% (with images)	-	-	p<0.001 (4.0 vs 3.5 with no images)<br><br>p=0.947 (1st vs 2nd entry for images on 4.0)<br><br>p=0.226 (2nd entry 4.0)<br><br>p=0.305 (2nd entry 3.5)
Fiedler et al. 2024	-	60.8% (text based)	66.7% (texted based)<br>53.2% (image based)<br>60.21% (overall)		76.4% (text based)<br>73.9% (image based)<br>75.3% (overall)	p =0.044 (residents vs 3.5 in text-based)<br><br>p=0.019 (residents vs 4.0 in image-based)<br><br>p=0.136 (residents vs 4.0 in text-based)<br><br>p=0.012 (residents vs 4.0 in all questions)<br><br>p=0.268 (4.0 vs 3.5 in text-based)
Hofmann et al. 2024	-	46.3% (overall)	63.4% (overall)	-	-	p<0.00001 (4.0 vs 3.5)
Lubitz et al. 2024	69.1% (overall)<br>77.8% (text describing media)	-	-	49.8% (overall)<br>58% (text describing images)	-	p<0.0001 for Bard w/ text description

Posner et al. 2024	-	53.4% (overall 1st entry)<br>50.1% (overall 2nd entry)	59.36% (overall 1st entry)<br>56.60% (overall 2nd entry)<br>67.81% (no images)<br>47.59% (with images)	-	-	p<0.001 (4.0 vs 3.5 with no images)<br><br>p=0.947 (1st vs 2nd entry for images on 4.0)<br><br>p=0.226 (2nd entry 4.0)<br><br>p=0.305 (2nd entry 3.5)
Fiedler et al. 2024	-	60.8% (text based)	66.7% (texted based)<br>53.2% (image based)<br>60.21% (overall)		76.4% (text based)<br>73.9% (image based)<br>75.3% (overall)	p =0.044 (residents vs 3.5 in text-based)<br><br>p=0.019 (residents vs 4.0 in image-based)<br><br>p=0.136 (residents vs 4.0 in text-based)<br><br>p=0.012 (residents vs 4.0 in all questions)<br><br>p=0.268 (4.0 vs 3.5 in text-based)
Hofmann et al. 2024	-	46.3% (overall)	63.4% (overall)	-	-	p<0.00001 (4.0 vs 3.5)
Lubitz et al. 2024	69.1% (overall)<br>77.8% (text describing media)	-	-	49.8% (overall)<br>58% (text describing images)	-	p<0.0001 for Bard w/ text description

Supplemental Table S1. Accuracy of LLM Answers to Orthopaedic Assessment Questions versus Orthopaedic Residents
