# Supplementary material for: Systematic Review on Large Language Models in Orthopaedic Surgery
Source: J Clin Med. 2025 Aug 20;14(16):5876. doi: 10.3390/jcm14165876 (PMC12386971; doi:10.3390/jcm14165876)
Supplement: Supplementary file 1 [file jcm-14-05876-s001.zip › LLM Supplemental Table S2.pdf]

Study Name:	ChatGPT 3.5	ChatGPT 4.0	p-value
Massey et al. 2023	37.8 % (no images)<br>22.4% (with images)<br>29.4% (overall)	61.0% (no images)<br>35.7% (with images)<br>47.2% (overall)	p<0.001<br>p<0.001 (3.5/4.0 vs Residents)<br>p=0.002 (4.0 vs 3.5)<br>p=0.033 (ChatGPT 3.5)<br>p<0.001 (ChatGPT 4.0)
Kung et al. 2023	54.3% (overall no images)	73.6% (overall no images)	-
Rizzo et al. 2023	50.24% (2022 overall)<br>47.42% (2021 overall)<br>46.51% (2020 overall)<br>46.34% (2022 with media)<br>42.31% (2021 with media)<br>38.37% (2020 with media)<br>52.80% (2022 without media)<br>52.29% (2021 without media)<br>51.94% (2020 without media)<br>38.30% (2022 first order)<br>50.00% (2021 first order)<br>53.70% (2020 first order)<br>53.75% (2022 higher order)<br>45.38% (2021 higher order)<br>39.25% (2020 higher order)	67.63% (2022 overall)<br>58.69% (2021 overall)<br>59.53% (2020 overall)<br>65.85% (2022 with media)<br>51.92% (2021 with media)<br>46.51% (2020 with media)<br>68.8% (2022 without media)<br>65.14% (2021 without media)<br>68.22% (2020 without media)<br>63.83% (2022 first order)<br>57.45% (2021 first order)<br>65.74% (2020 first order)<br>68.75% (2022 higher order)<br>59.66% (2021 higher order)<br>53.27% (2020 higher order)	-
Nakajima et al. 2024	28, 32, 30% (all questions)<br>33, 27, 18% (without images)<br>30% (overall)	60, 55, 61% (all questions)<br>64, 63, 73% (without images)<br>59% (overall)	p < 0.001 (4.0 vs 3.4 all questions)<br>p=0.002 (text based)
Posner et al. 2024	53.4% (overall 1st entry)<br>50.1% (overall 2nd entry)	59.36% (overall 1st entry)<br>56.60% (overall 2nd entry)	p<0.001 (4.0 vs 3.5 with no images)

		67.81% (no images)<br>47.59% (with images)	p=0.947 (1st vs 2nd entry for images on 4.0)<br><br>p=0.226 (2nd entry 4.0)<br><br>p=0.305 (2nd entry 3.5)
Fiedler et al. 2024	60.8% (text based)	66.7% (texted based)<br>53.2% (image based)<br>60.21% (overall)	p=0.268 (4.0 vs 3.5 in text-based)
Hofmann et al. 2024	46.3% (overall)	63.4% (overall)	p<0.00001 (4.0 vs 3.5)
Agharia et al. 2023	40.2% (picked popular response orthobullets)	68.0% (picked popular response orthobullets)	p<0.001
Fahy et al. 2024	DISCERN: 55.4<br>Flesh-Kincaid: 14.7	DISCERN: 62.09<br>Flesh-Kincaid: 13.7	-
Mejia et al. 2024	Accuracy to NASS: 52%<br>Overinclusive: 48%<br>Incomplete response: 38%	Accuracy to NASS: 59%<br>Overinclusive: 45%<br>Incomplete response: 28%	p=0.792 (accuracy)<br>p=1.000 (overinclusive)<br>p=0.313 (completeness)
Zaidat et al. 2024	Accuracy to NASS: 62.5%<br>Inaccurate: 37.5%	Accuracy to NASS: 81%<br>Inaccurate: 19%	-

Supplemental Table S2. Comparison of ChatGPT 3.5 vs ChatGPT 4.0
