# Supplementary material for: Systematic Review on Large Language Models in Orthopaedic Surgery
Source: J Clin Med. 2025 Aug 20;14(16):5876. doi: 10.3390/jcm14165876 (PMC12386971; doi:10.3390/jcm14165876)
Supplement: Supplementary file 1 [file jcm-14-05876-s001.zip › LLM Supplemental Table S3.pdf]

Articles	LIKERT scale	DISCERN	Flesh-Kincaid	Recommended Guidelines	p-values
Draschl et al. 2023	3.87/5 (ChatGPT 3.5)	-	-	-	p<0.001
Hurley et al. 2024	-	60 (ChatGPT)	26.2	-	-
Subramanian et al.	4.1/5 (ChatGPT)	-	11.2	-	-
Zhang et al.	4.9/5 relevance<br>4.6/5 factual accuracy (ChatGPT)	-	-	-	-
Johns WL, Martinazzi BJ et al.	-	41 (ChatGPT)	12	-	-
Wright et al.	-	-	11.65 (ChatGPT)	-	-
Fahy et al. 2024	-	GPT 3.5=55.4<br>GPT 4.0= 62.09	18.08 (ChatGPT 3.5)<br>17.9 (ChatGPT 4.0)	-	p<0.01 (DISCERN)<br>p=0.95 (Flesh-Kincaid
Nian et al.	-	-	11.6 (ChatGPT)	-	-
Johns WL, Kellish A et al.	-	-	11.51	-	-
Zaidat et al. 2024	-	-	-	NASS<br>3.5- 10/16 (62.5%)<br>4.0- 13/16 (81.25%)	-
Mejia et al. 2024	-	-	-	NASS<br>3.5- 15/29 (51.7%)<br>4.0- 17/29 (58.6%)	p=0.792
Yang et al.	-	-	-	AAOS CPG<br>16/20 (80%; ChatGPT)<br>12/20 (60%; Bard)	p=0.34
Chalhoub et al.	-	-	-	ChatGPT 70% accuracy rate for diagnosis, 95% for management recommendations	-
Shrestha et al.	-	-	-	NASS	p=0.41 (sufficient data)

				ChatGPT 65% accurate for sufficient data<br>46% accurate for insufficient data<br>49% accurate for no adequate studies	p=0.11 (insufficient data)<br>p=0.003 (no adequate studies)
Parekh et al. 2024	-	-	-	46.2% (AOFAS ChatGPT)<br>36.5% (Bard)	-

Supplemental Table S3. LLM performance on Patient Questions
